# Supplementary material for: Suitability of Nanoparticles to Face Benzo(a)pyrene-Induced Genetic and Chromosomal Damage in M. galloprovincialis. An In Vitro Approach
Source: Nanomaterials (Basel). 2021 May 15;11(5):1309. doi: 10.3390/nano11051309 (PMC8155950; doi:10.3390/nano11051309)
Supplement: Supplementary file 1 [file nanomaterials-11-01309-s001.zip › Figure S1.pdf]

A

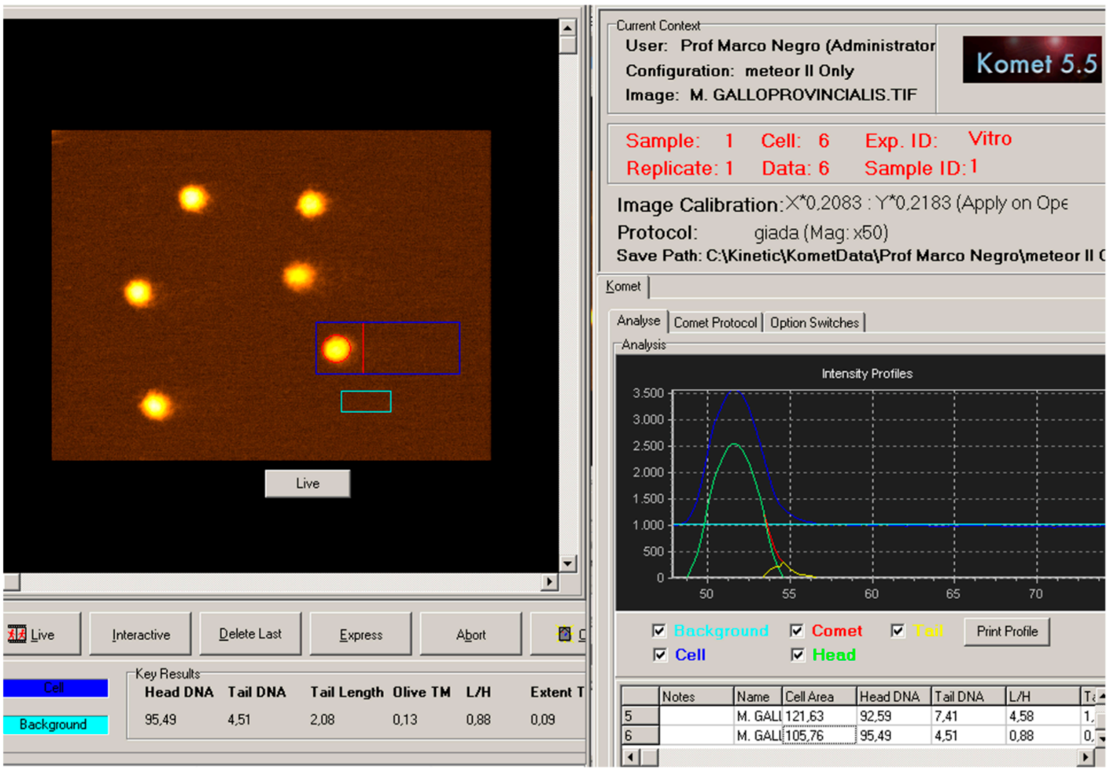

B

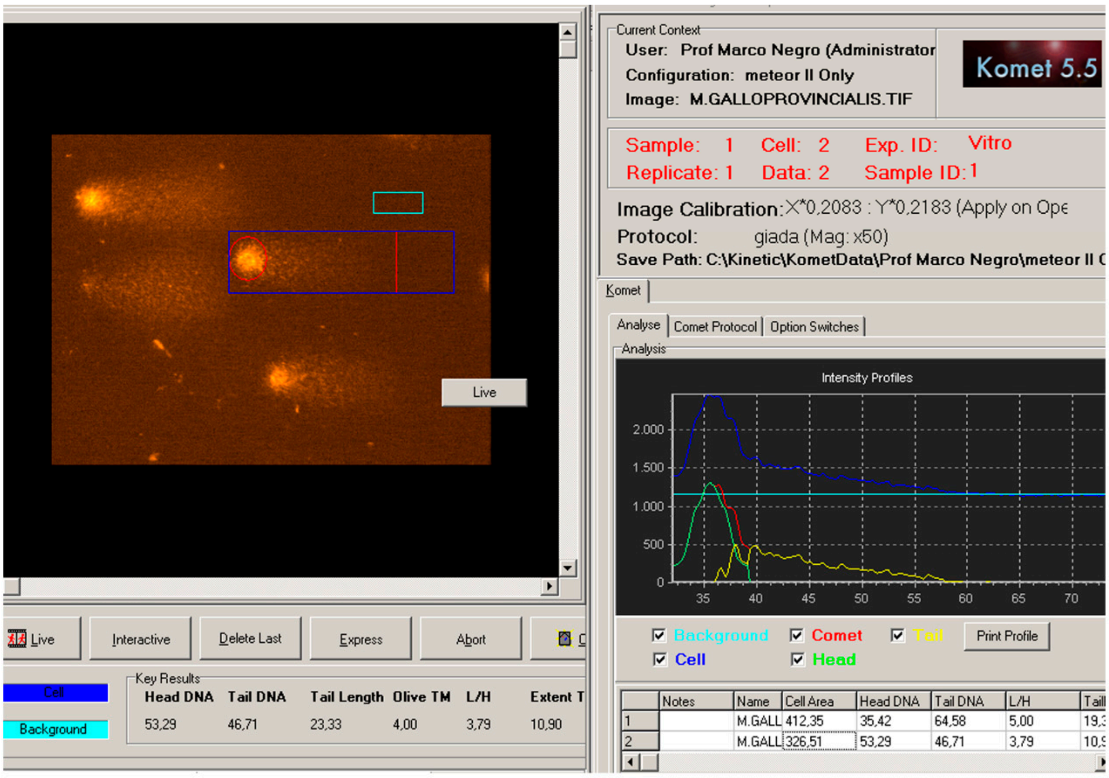

Figure S1: Comet assay evaluation: DNA damage was evaluated as the percentage of DNA migrating out of the nucleus by an image analyzer (Komet 5.0 Software, Kinetic Imaging Ltd) connected to the fluorescent microscope. Tail DNA (%) was chosen as a reliable Comet assay parameter. 9 mussels per treatment group, 2 slides per mussel were setup and 50 random nuclei *per slide* were scored and the mean was calculated. A) an

example of randomly scored undamaged nuclei from control cells. B) an example of randomly scored damaged nuclei from B(a)P exposed cells.
