# Supplementary material for: Suitability of Nanoparticles to Face Benzo(a)pyrene-Induced Genetic and Chromosomal Damage in M. galloprovincialis. An In Vitro Approach
Source: Nanomaterials (Basel). 2021 May 15;11(5):1309. doi: 10.3390/nano11051309 (PMC8155950; doi:10.3390/nano11051309)
Supplement: Supplementary file 1 [file nanomaterials-11-01309-s001.zip › Figure S2.pdf]

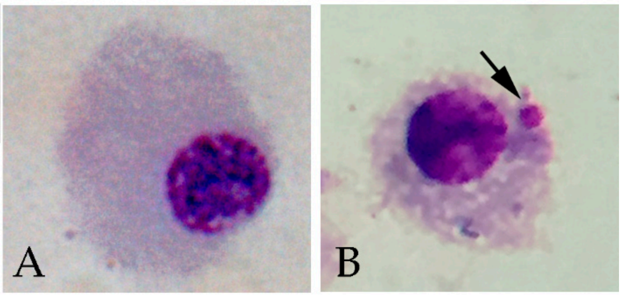

Figure S2. Micronuclei scoring: images obtained by optical microscopy (100x) of M. galloprovincialis gills cells. 9 mussels *per* treatment group, 2 slides *per* mussel were setup, 500 random gill cells with well preserved-cytoplasm *per* slide were scored and the mean calculated A: Mononucleated healthy cell; B: gill cell displaying a micronucleus (MN), indicated by an arrow.
