# Supplementary material for: Suitability of Nanoparticles to Face Benzo(a)pyrene-Induced Genetic and Chromosomal Damage in M. galloprovincialis. An In Vitro Approach
Source: Nanomaterials (Basel). 2021 May 15;11(5):1309. doi: 10.3390/nano11051309 (PMC8155950; doi:10.3390/nano11051309)
Supplement: Supplementary file 1 [file nanomaterials-11-01309-s001.zip › Figure S3.pdf]

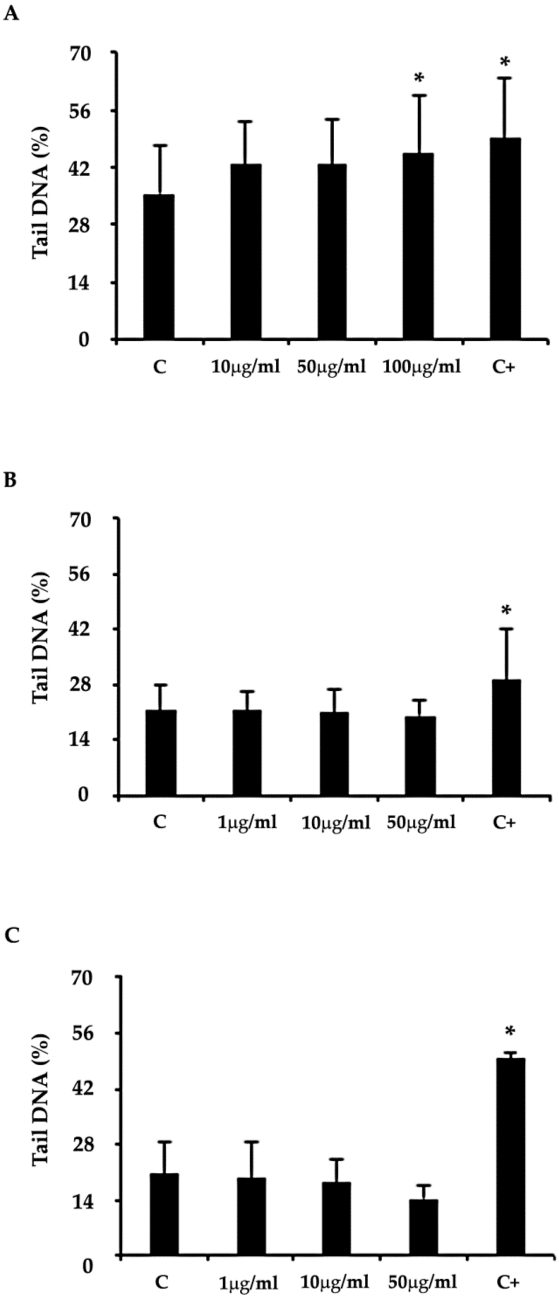

Figure S3: Levels of DNA damage in gill cells of *M. galloprovincialis* after in vitro exposure to different concentrations of NPs. *$p < 0,05$ (MANOVA). A: Hydrophilic CB-derived nanoparticles (HNP). B: P25. C: MT.
