# Supplementary material for: Suitability of Nanoparticles to Face Benzo(a)pyrene-Induced Genetic and Chromosomal Damage in M. galloprovincialis. An In Vitro Approach
Source: Nanomaterials (Basel). 2021 May 15;11(5):1309. doi: 10.3390/nano11051309 (PMC8155950; doi:10.3390/nano11051309)
Supplement: Supplementary file 1 [file nanomaterials-11-01309-s001.zip › Table S1.pdf]

Table S1: Main articles cited in the discussion section. SWCN: Single Walled Carbon Nanotubes; MWCN: Multiple Walled Carbon Nanotubes; HNP: carbon black (CB)-derived hydrophilic NPs; CB: Carbon Based NPs. (+) indicates biological effects exerted by the tested NPs; (-) indicates the absence of biological effects exerted by the tested NPs.

Substance	Test	Experimental Model	Biological effects	Reference
**HNP**	*In vivo* embryo-toxicity and survival	*D. rerio*	(-) No toxicity and high survival rate	[59]
**SWCNT**	Mytotic spindle and centrosome morphology analysis. FISH. Cell viability and apoptosis.	SAEC, BEAS-2B	(+) Mitotic spindle distruption and errors in chromosome number	[60]
**SWCN and MWCNT**	Micronucleus test	RAW 264.7	(+/-) ROS release, necrosis and chromosomal aberrations. Did not cause inflammatory response	[61]
**SWCN and MWCNT**	Comet assay and Micronucleus test	RAW 264.7	(+) Genotoxic and cytotoxic effects	[63]
**CB NPs**	Micronucleus test, MTT assay, ELISA, Trypan Blue	RAW 264.7	(+) Cell proliferation inhibition, MN induction, increased apoptosis	[64]
**Rutile and Anatase TiO_2 NPs**	BAL fluid inflammatory markers, cell proliferation, histopathology	*In vivo* rats	(-) lung toxicity of anatase/rutile should not be viewed as representative for all ultrafne TiO_2 particles types.	[66]
**Rutile and Anatase TiO_2 NPs**	Colony forming efficiency assay, cell transformation assay and the micronucleus test	Balb/3T3	(+/-) dose-dependent reduction of the clonogenic activity, rutile TiO_2 NPs were able to induce type-III foci formation. anatase TiO_2 NPs did not induce any significant neoplastic or genotoxic effect.	[68]
**TiO_2 NPs**	Comet assay, hystopathology, gene expression, oxidative DNA damage	*M. galloprovincialis* haemocytes	(+) Genotoxic effects induced by oxidative stress	[69]
**Rutile TiO_2 NPs**	Embryo acute toxicity test, adult toxicity test, ELISA, qRT-PCR.	*D. rerio*	(+) TiO_2 NPs can cause oxidative damage to organisms	[70]
**Rutile and Anatase TiO_2 NPs**	Comet assay	dolphin leukocytes, human leukocytes, 3T3, HuDE	(+) Increased DNA damage in bottlenose dolphin leukocytes and 3T3 after rutile exposure<br>(+) human fibroblasts showed a significant dose–response effect exposure to anatase.<br>(-) Human leukocytes were tolerant to both anatase and rutile.	[71]
**TiO_2 NPs**	Eco-toxicity test	*B. subtilis*, *E. coli*	(+) bacterial growth inhibition	[72]
